# New Insights into Glaucoma—An Editorial Review on Broadening Risk Assessment, Refining Surgical Strategy, and Standardizing Clinical Practice

**DOI:** 10.3390/jcm15031213

**Published:** 2026-02-04

**Authors:** Su-Ho Lim, Daniel Laroche

**Affiliations:** 1 Hamilton Glaucoma Center, Shiley Eye Institute, Viterbi Family Department of Ophthalmology, University of California San Diego, La Jolla, CA 92037, USA; 2 Department of Ophthalmology, Daegu Veterans Health Service Medical Center, Daegu 42835, Republic of Korea; 3 Advanced Eyecare of New York, New York, NY 10027, USA

Glaucoma remains a major leading cause of irreversible blindness worldwide, with disease progression influenced by complex interactions, such as intraocular pressure (IOP), optic nerve head structure, vascular factors, multiple systemic diseases, and long-term treatment tolerability [1,2]. While lowering IOP remains the only proven strategy to slow disease progression, effective glaucoma care in practice is affected by various factors including accurate IOP measurement, treatment adherence, the management of coexisting ocular diseases, the appropriate selection of surgical options, and postoperative management [1,2]. In this context, this Special Issue of *Journal of Clinical Medicine*, “New Insights into Glaucoma” brings together ten studies that address these challenges across diagnostics, medical therapy, and surgery, offering pragmatic, clinically relevant evidence to inform and guide everyday decision-making.

## 1. Introduction

Glaucoma is a progressive optic neuropathy with highly variable clinical trajectories, even among patients with similar IOP levels and structural damage [1,2]. Large clinical trials and population-based studies have clarified the advantage of IOP reduction and identified risk factors including systemic, vascular, and treatment-related elements beyond IOP control alone [1,3,4]. The contributions in this Special Issue build on this foundation by highlighting how the measurement context, ocular surface health, affordability, surgical technique, lens extraction, affordable microinvasive glaucoma surgery, and systemic comorbidity can meaningfully influence outcomes over a patient’s lifetime.

## 2. Measurement Matters: IOP as a Contextualized Parameter

IOP remains the cornerstone of glaucoma management; however, its interpretation is highly dependent on measurement conditions [2,3,4]. In routine clinical practice, IOP is frequently measured after visual field testing without considering the potential short-term physiological effects. Jang et al. (Special Issue) directly addressed this issue by demonstrating that automated visual field testing is associated with measurable changes in IOP when comparing pre- and post-visual field test measurements (DOI: 10.3390/jcm14186356). This finding is consistent with previous reports showing that perimetric testing itself can influence short-term IOP behavior [5] and has clear practical implications.

Measurement challenges are even more pronounced in pediatric glaucoma and eyes with anterior segment abnormalities. Studer et al. (Special Issue) compared Goldmann applanation tonometry, iCare PRO, and Tono-Pen measurements under general anesthesia in young children (DOI: 10.3390/jcm14103338). Their results highlight device-dependent differences that may affect longitudinal assessments, reinforcing the need for consistency in tonometry modalities and cautious interpretation of absolute IOP values in pediatric care.

## 3. Glaucoma Medication, Ocular Surface Health, and Sustainability

Long-term glaucoma medical therapy remains central to glaucoma care. However, its effectiveness is limited by ocular surface toxicity and poor adherence [6]. Preservatives such as benzalkonium chloride have been shown to induce inflammation, discomfort, and reduced quality of life, which in turn compromise treatment persistence and surgical readiness [1,6].

In this context, Kim et al. (Special Issue) conducted a randomized clinical trial to compare preserved and preservative-free fixed combinations of brimonidine and timolol (DOI: 10.3390/jcm14051587). Although the IOP-lowering efficacy was comparable, the preservative-free formulation demonstrated superior ocular surface outcomes and improved tolerability compared with the preservative-containing formulation in their study. Clinically, this study supports a shift toward treatment strategies that prioritize long-term sustainability over short-term pressure reduction alone, while also reminding us of the importance of surgery in reducing medication burden and the potential lifelong side effects associated with chronic topical therapy

## 4. Surgical Accessibility and Equity: Low-Cost but Effective Options

As the global burden of glaucoma has increased, disparities in access to surgical care have become increasingly evident. Calderon and Laroche et al. (Special Issue) addressed this challenge by evaluating a low-cost surgical approach, the Sinskey hook goniotomy combined with cataract surgery, in Black and Afro-Latino patients with glaucoma (DOI: 10.3390/jcm14103266). At one year postoperatively, clinically meaningful IOP lowering and a significant reduction in medication burden were achieved, with a high proportion of patients remaining medication-free. This study expands on previous work on angle-based minimally invasive glaucoma surgery by demonstrating that an effective glaucoma intervention does not always require expensive disposable devices [7,8]. Its inclusion in this Special Issue highlights its affordability as a legitimate dimension of surgical innovation and underscores the importance of aligning technique selection with population-specific needs, an especially important consideration for patients in resource-poor areas globally.

## 5. Surgical Outcomes: Techniques, Devices, and Preoperative Evaluation

Surgical success in glaucoma depends not only on the choice of procedures but also on tissue characteristics and perioperative management [9]. Several studies in this Special Issue illustrate how incremental refinements can improve outcomes. Yamazaki et al. (Special Issue) investigated whether preoperative exposure to prostaglandin analogs with differing potentials for prostaglandin-associated periorbitopathy (PAP) influences outcomes after trabeculectomy and Ahmed glaucoma valve implantation (DOI: 10.3390/jcm14196940). Their findings suggest that chronic topical glaucoma medication may affect periocular tissue response and relative surgical success, reinforcing the need to consider preoperative glaucoma medication history during surgical planning.

Device-based surgery is addressed in two complementary articles in this Special Issue. Kim et al. (Special Issue) present two-year outcomes of ab interno XEN45 gel stent implantation in open-angle glaucoma (DOI: 10.3390/jcm14134617), demonstrating sustained IOP reduction despite frequent early hypotony that generally resolved over time. In pseudoexfoliation glaucoma, Gehrke et al. (Special Issue) showed that temporary intraluminal nylon stenting during PRESERFLO MicroShunt implantation reduced early hypotony-related complications (DOI: 10.3390/jcm14176224). These studies highlight how postoperative safety can be improved through thoughtful modulation of aqueous outflow rather than wholesale changes in surgical strategy. In addition, a long-term perspective is essential. Fiore et al. (Special Issue) report five-year outcomes of deep sclerectomy in pseudoexfoliation versus primary open-angle glaucoma (DOI: 10.3390/jcm13237434), providing valuable diagnosis-specific data that inform patient counseling and long-term follow-up strategies.

## 6. Systemic and Sensory Dimensions of Glaucoma

Growing evidence supports a broader view of glaucoma as a condition influenced by systemic vascular and cardiometabolic factors rather than an isolated ocular disorder [1,2,3,4]. In this context, Lee and Seo et al. (Special Issue) employed a two-sample Mendelian randomization approach to investigate the relationship between atrial fibrillation/flutter and primary open-angle glaucoma (DOI: 10.3390/jcm13247670). Using genetic variants as instrumental variables, this study minimized the residual confounding and reverse causation inherent in conventional observational analyses and provided evidence supporting a potential causal association between atrial fibrillation/flutter and glaucoma. These findings extend previous epidemiological observations and underscore the clinical relevance of cardiovascular risk profiling and interdisciplinary collaboration for glaucoma care, while highlighting the importance of reinforcing healthy diet and exercise habits in our patients to maintain optimal cardiovascular health.

Beyond vascular comorbidity, Meliante et al. (Special Issue) explored the relationship between asymmetric glaucoma and ipsilateral hearing impairment (DOI: 10.3390/jcm13216501). Although exploratory, their findings raise clinically relevant questions regarding multisensory dysfunction and quality-of-life considerations, particularly in patients with unilateral or asymmetric diseases.

## 7. Concluding Perspective

The studies featured in this Special Issue collectively advance our understanding of glaucoma as a multifactorial disease influenced by ocular, systemic, and socioeconomic variables. Together, they emphasize that individualized care grounded in context-specific measurement, ocular surface preservation, surgical precision, and affordability can deliver sustained benefits over a patient’s lifetime. By exploring innovations such as affordable microinvasive glaucoma surgery, combined with lens extraction, these contributions demonstrate that surgical success need not depend on costly instrumentation but rather on thoughtful adaptation to clinical contexts and patient needs, particularly in resource-limited regions.

Equally important, the findings underscore that comprehensive glaucoma management extends well beyond IOP reduction. The integration of systemic risk assessment, cardiovascular health promotion through diet and exercise, and a reduction in medication-related burden through timely surgical intervention represent a truly multidisciplinary approach. As this body of work shows, sustainable glaucoma care in the modern era relies on aligning technological innovation, clinical insights, and global health equity to secure meaningful, lifelong outcomes for patients across diverse populations. By integrating established evidence from prior publications, this Special Issue offers personalized, long-lasting, and equitable clinically relevant insights into glaucoma management.

## Abbreviations

The following abbreviations are used in this manuscript:

IOP	Intraocular pressure
